# Healthy universities: Exploring the relationship between psychosocial needs and work-related health among university employees

**DOI:** 10.1080/15555240.2023.2194026

**Published:** 2023-03-18

**Authors:** S. T. Innstrand, C. Banks, C. Maslach, C. Lowenstein

**Affiliations:** a Department of Psychology, Norwegian University of Science and Technology, Trondheim, Norway; b Interdisciplinary Center for Healthy Workplaces, University of California, Berkeley, Berkeley, California, USA; c Department of Epidemiology and Population Health, Stanford University, Stanford, California, USA

**Keywords:** Gender, healthy university, multigroup SEM, needs

## Abstract

The present study explores psychosocial needs among university employees and the extent to which these needs influence employee perceptions of how work positively or negatively affects their health. Structural equation modeling (SEM) analyses among Norwegian faculty members (*N* = 11,533) suggest that needs differ in importance to the two work-related health outcomes. Multi-group analyses suggest gender differences in the level of these needs and in their degree of relationship with positive/negative work-related health. Among women, the strongest predictors of positive and negative work-related health are work engagement and autonomy, respectively. Among men, the strongest predictors of positive and negative work-related health are meaning and social community, respectively. Although significant differences were found in the level of the psychosocial needs across different university groups (faculty, PhD students, administrative/technical staff), their predictive value for how work affects their health positively or negatively is basically equivalent across groups. Study findings raise two implications: (1) the mechanisms and characteristics of the work environment that promote *versus* detract from health in the university setting do not appear to be two sides of the same coin and suggest different sets of interventions for improving employee health, and (2) gender differences should be taken into account in designing interventions to improve health and well-being in universities.

## Introduction

The interest in healthy universities has expanded alongside a growing awareness that the university as a health-promoting setting is essential not only for the members of its community but also for a sustainable society (Innstrand & Christensen, 2020). As intellectual capital is a university’s primary and only appreciable asset, faculty members’ motivation, commitment, and well-being are critical for the ability of educational institutions to fulfill their mission. Yet, there is a lack of knowledge of what promotes health and well-being at the university.

Our study builds on need theory, which posits that people are driven to satisfy their basic needs, and when these needs are satisfied, they experience well-being. Conversely, when satisfaction of needs is thwarted or non-existent, people experience stress and frustration (Deci & Ryan, 2000). Need satisfaction is viewed as an essential nutrient for optimal functioning across individual differences and cultures (Chen et al., 2015). For example, need theories have proven useful in explaining how intrinsic goal framing produces vibrant learning environments for students (Vansteenkiste, Lens, & Deci, 2006) and greater receptivity toward changing teaching strategies for physical education (PE) teachers (Aelterman, Vansteenkiste, Van Keer, & Haerens, 2016). Although studies exist that examine the role of needs in organizational functioning, little research has focused on the relationship between psychosocial needs and health and well-being for different groups of employees in academic settings.

The World Health Organization defines health both positively as complete physical, mental, and social well-being, and not merely negative as the absence of disease or infirmity (Grad, 2002, p. 981). Previous findings support a two-dimensional structure of health and suggest that psychological distress and subjective well-being are distinct and complementary constructs and not merely two poles of the same continuum (Winzer, Lindblad, Sorjonen, & Lindberg, 2014). As low-level psychological distress does not mean automatically high subjective well-being it has been recommended to use concomitant measures of positive and negative manifestations (Massé et al., 1998). Taking this into consideration, the present study utilizes a two-dimensional structure of health related to work labeled negative work-related health and positive work-related health, respectively. Thus, the present study expands previous findings by exploring group-specific differences in the level of different psychosocial needs and how they relate to both positive and negative work-related health in a university setting.

### Theoretical framework

There has been substantial research on how satisfaction of basic needs enhances employee motivation since Maslow’s seminal work on the hierarchy of needs (Maslow, 1943). One frequently cited framework on psychological needs is the Self-Determination Theory (SDT) (Deci & Ryan, 2000), which identifies three basic psychological needs: competence, relatedness, and autonomy. According to this theory and widely supported, satisfaction of these needs leads to ongoing psychological growth, integrity, and well-being, and conditions that thwart need satisfaction can lead to degradation of well-being or ill-being (Deci & Ryan, 2000; Olafsen, Niemiec, Halvari, Deci, & Williams, 2017; Van den Broeck, Ferris, Chang, & Rosen, 2016). Intrinsic motivation and basic need satisfaction also facilitates effective performance and well-being in particular if the tasks are more heuristic and require creativity, cognitive flexibility, or deep processing of information (Gagne & Deci, 2005) and thus, is directly relevant to to knowledge work and hence, academia.

Although the SDT has a strong empirical foundation, there is a lack of studies that have tested the theory within different organizational settings (Gagne & Deci, 2005) and university settings in particular. In a meta-analytic review of studies on basic need satisfaction, Van den Broeck et al. (2016) called for more exploratory research comparing SDT’s basic psychological needs to other potential needs and motivational constructs. One such study identified seven different needs, or states, that have the potential to boost work motivation and well-being: autonomy, belongingness, competence, psychological safety, positive emotions, fairness, and meaning (Maslach & Banks, 2017).

Gappa and Austin (2010) described different challenges facing higher education institutions today and, argued that faculty members’ essential needs go beyond academic freedom, shared governance, and job security. They added equity, collegiality, flexibility, and professional growth. Joined by respect, which is seen as the core value on which all others rest, this extended list constitutes the “Essential Elements” of twenty-first-century faculty work and pertain to all faculty members regardless of their appointment type. They argue that these elements are significant for attracting and retaining excellent academic employees in times of change.

Informed by the seven needs identified by Maslach and Banks (2017) and by the essential elements identified for faculty members in the twenty-first century (Gappa & Austin, 2010), we explored how different needs related to health and well-being in academia. Specifically, we explored the predictive value of *job autonomy*, *social community*, *task completion clarity*, *work engagement*, *trust in unit management*, *recognition*, and *meaning* on *work-related positive and negative health* among university employees.

### Psychosocial needs in higher education

Autonomy and academic freedom are seen as the core of traditional academic value (Aberbach & Christensen, 2018), a primary intrinsic motivator among academics (Bellamy, Morley, & Watty, 2003), and a basic need for health and well-being (Maslach & Banks, 2017). In addition, Gappa and Austin (2010) argue that changes in societal and faculty expectations of work and worklife issues compel the need for balance and flexibility in their career. Whereas job autonomy refers to perceived control over schedules and the organization of work, academic freedom refers to academic staff’s empowerment to make decisions regarding teaching and research, the core activities and tasks of the university (Aberbach & Christensen, 2018). In the present study, we measure autonomy by respondents’ influence over how the work is carried out and academic freedom by their ability to determine when their tasks are completed (task completion clarity). Thus, we hypothesized that;

Hypothesis 1. Higher levels of perceived (a) job autonomy, and (b) task completion increases employees’ perception that work influences their health positively
Hypothesis 2. Higher levels of perceived (a) job autonomy, and (b) task completion decreases employees’ perception that work influences their health negatively

It has been argued that psychological safety in teams (PST), characterized by *interpersonal trust*, *respect*, and *caring within the work team*, is a prerequisite for learning (Edmondson, 1999) and, health and well-being (Maslach & Banks, 2017). Because of their relevance to academics, we explored how these three characteristics relate to work-related health in academic settings. First, increasing diversity in employee demographics, such as gender, ethnicity and race, family status, and age, demand the need for trust (Gappa & Austin, 2010). For example, Downey, van der Werff, Thomas, and Plaut (2015) found that a trusting climate directly related to inclusion and mediated the positive effects of diversity practices on work engagement. Based on their findings, they call for more studies exploring how trust at different levels (e.g., trust in supervisor, team trust, trust in organization) are related to employee well-being. Therefore, we explored how psychological safety, as measured by the variable, *trust in unit management*, relates to employee work-related health.

Second, fairness or equity is considered highly significant for employee health and well-being in general (Maslach & Banks, 2017) and among academics in particular (Gappa & Austin, 2010). Fairness relates to the extent that decisions at work are perceived as just, and people are treated with respect. In the present study the need for fairness and respect (i.e., I am respected by the unit management, I am treated fairly by the unit management) are measured by the variable, *recognition*. A Norwegian study among academics found that recognition is particularly important among older workers, predicting meaning and commitment at work (Anthun & Innstrand, 2016). However, many academics report that they are not valued or recognized for their work (Bellamy et al., 2003). Thus, lack of satisfaction with this need could generate a possible hazard for academics’ health and well-being as well as their organizational commitment.

Third, caring within the work teams, or collegiality, is also identified as one of the essential needs for faculty members (Gappa & Austin, 2010) and is closely related to the SDT need, relatedness, or alternately labeled belongingness (Maslach & Banks, 2017). Relatedness is the need to feel connected to others and is satisfied when people see themselves as a member of a group, experience a sense of communion, and develop close relations. Although past work on SDT argued that relatedness plays a more distal role than autonomy or competence, a recent meta-analysis showed that relatedness plays a more significant role than previously expected and is strongly related to health and well-being (Van den Broeck et al., 2016). In the present study, this need is operationalized and measured by social community at work.

Hypothesis 3. Higher levels of perceived (a) trust in unit management (b) recognition, and (c) social community increases employees’ perception that their work influences their health positively
Hypothesis 4. Higher levels of perceived (a) trust in unit management (b) recognition, and (c) social community decreases employees’ perception that their work influences their health negatively

Lastly, professional growth is suggested to be an essential element that needs to be present in faculty members’ working environment to recruit and retain faculty members (Gappa & Austin, 2010). Within the SDT, psychological growth is typically manifested by intrinsic motivation where engagement in work is considered enjoyable in and of itself (Van den Broeck et al., 2016). *Work engagement*, which is defined as a positive work-related state of mind characterized by the feelings of vigor, dedication, and absorption (Schaufeli, Salanova, Gonzalez-Romá, & Bakker, 2002), can be interpreted as intrinsic motivation. Work engagement is particularly important in knowledge intensive work-places, such as universities and colleges, where the employees are the primary bearers of knowledge and thus, the competitive parameter in those organizations (Ipsen & Jensen, 2012). Several studies have found that work engagement is linked to both improved job performances, psychological well-being, and health in academia (Christensen, Dyrstad, & Innstrand, 2020; Innstrand, Christensen, & Helland, 2022).

Work engagement is also closely related to *meaning*, which has positive associations with health and well-being (Maslach & Banks, 2017). However, meaning can differ from work engagement by arising in “transcendent moments in time,” rather than as a sustained state of being (Bailey & Madden, 2017). According to Bailey and Madden (2017) are these moments “imbued with a sense of the coming together of the practice of work, reflection on that practice and the sense of a job well done, connection with others, coupled with an awareness of the significance of work in its historical and future context” (p. 15). Further they claim that meaning can arise “when an individual perceives an authentic connection between their work and a broader transcendent life purpose beyond the self” (p. 4). Using SDT terminology, meaning might be defined as a motivation in which behavior is more self-endorsed and viewed as important and/or in line with one’s closely held values (Van den Broeck et al., 2016). Meaning in work is highly significant for academics, and despite worsening work conditions in the academic sector, many academics consider their position as a calling, not just a job (Bellamy et al., 2003). Therefore, meaning in work is not only positively associated with health, but may act as a buffer against stress in one’s work and work life. In the present study, both work engagement and meaning are hypothesized to relate to university employees’ work-related health:

Hypothesis 5. Higher levels of perceived (a) work engagement, and (b) meaning increases employees’ perception that work influences their health positively
Hypothesis 6. Higher levels of perceived (a) work engagement, and (b) meaning decreases employees’ perception that work influences their health negatively

### Individual differences

Individual differences in psychosocial needs can be found in mean levels and in the strength of relationships in the hypothesized model presented here. Although the benefits associated with need satisfaction are universal, the emphasis on these needs might differ in accordance with the values and practices of different cultural climates, as suggested by Vallerand (2000). Differences in the cultural climate and satisfaction of needs does not only relate to cultural diversity across countries but might also exist across work environments due to differences in opportunities or resources for need satisfaction, variation in the emphasis placed on the needs, or in the way these needs are met (Chen et al., 2015). As such, the level of needs might vary across different groups.

Chen et al. (2015), however, found the relation between need satisfaction and well-being to be invariant across cultures and not moderated by need strength. This suggests that basic needs contribute to well-being regardless of the extent to which needs are valued. If so, this supports the universality assumption central to SDT, conceptualizing needs as necessities for psychological well-being rather than socially constructed preferences (Gagne & Deci, 2005). The present study explores both the invariance of the level of the needs as well as in its relationship with positive/negative work-related health by means of multi-group analyses across academic groups (administrative staff, PhD students, and faculty), and gender. Based on the assumption by SDT and previous findings, we hypothesized that:

Hypothesis 7. (a) The *level* of the psychosocial needs would be diverse across different academic groups (faculty, PhD students, technical/administrative staff) and gender (male, female)
(b) The *strength* of the association between the psychosocial needs and positive/negative work-related health would be invariant across academic groups (faculty, PhD students, technical/administrative staff) and gender (male, female)

## Methods

### Sample

Data for the present study were gathered from ARK,^1^ a healthy university initiative originated in Norway, that involved the administration of the KIWEST (Knowledge Intensive Work Environment Survey) to all faculty, administrative staff, and production workers in some of the largest Norwegian colleges and universities (Innstrand & Christensen, 2020). From autumn 2013 to spring 2015, all employees from the participating universities in ARK at that time, and with a regular payroll for a minimum of 20% position, were invited to answer the survey (*N* = 18,599). In total 12,170 responded, providing a response rate of 65%. In the present study, leaders were omitted from the analyses due to leadership questions. Thus, the final sample consisted of 4562 faculty (research and teaching), 1452 PhD students, and 5519 administrative and technical staff (*n* = 11,533). The sample was equally distributed with 54% women and 46% men. Age distributed as follows; under 30 years (9.8%), 30–39 years (23.2%), 40–49 years (27.2%), 50–59 years (24.3%), and 60 years or older (15.5%). Most were permanent employees (75%) and had a full-time position (86%). The demographics across the three employee categories are provided in Table 1. Due to concerns for anonymity, the universities did not provide data that identified to which faculties and departments the individual respondents belonged. The ARK study is approved by the Norwegian Center for Research Data (NSD) and informed consent was obtained by participants ticking “submit” at the end of the survey.

### Variables

All variables were from the KIWEST questionnaire, which was based on previously validated and tested scales measuring the psychosocial work environment (Innstrand, Christensen, Undebakke, & Svarva, 2015). All but the engagement and work-related health survey items had a response format ranging from 1 (*Strongly disagree*) to 5 (*Strongly agree*). In the engagement scale, the response alternatives ranged from 0 (*Never*), 1 (*A few times a year or less*), 2 (*Once a month or less*), 3 (*A few times a month*), 4 (*Once a week*), 5 (*A few times a week*), 6 (*Every day*), and in work-related health, the response alternatives ranged from 1 (*To a very small extent)* to 5 *(A very large extent*).

*Job autonomy* was measured by a four-item scale from Näswall et al. (2010) and measures the extent of autonomy and influence over how the work is carried out. Sample item: “I have a sufficient degree of influence in my work.”

*Task completion clarity* was measured by three items from Näswall et al. (2010) which aim to capture the extent to which the employees themselves can, or have to, determine when their tasks are completed. Sample item: “I determine when my work assignments are completed.”

*Trust in unit management* was measured by five items from Näswall et al. (2010) and reflects perceptions of the employers’ reliability and trust-worthiness. Sample item: “My unit management is always reliable.”

*Recognition* was measured by a three-item scale from COPSOC II (Pejtersen, Kristensen, Borg, & Bjorner, 2010), which assessed the extent respondents feel they are recognized and appreciated for their efforts. Sample item: “My work is recognized and appreciated by the unit management.”

*Social community* was measured by a three-item scale from COPSOQ II (Pejtersen et al., 2010), which assessed the extent respondents experience a strong degree of social community with colleagues in their own unit. Sample item: “There is a good atmosphere between me and my colleagues.”

*Work engagement* was measured by the shortened, nine-item version of the Utrecht Work Engagement Scale (UWES). This shortened version has shown satisfactory psychometric properties (Schaufeli, Bakker, & Salanova, 2006), and has been validated in many countries. Sample items: “At my work, I feel bursting with energy” (vigor); “I am proud on the work that I do” (dedication); and “I get carried away when I’m working” (absorption).

*Meaning of work* was measured by three items from COPSOQ I and II (Pejtersen et al., 2010) and captures respondents’ experience of having a meaningful job. Sample item: “My work is meaningful.”

*Work-related health* was measured by two single items assessing respondents’ experience of how the work impacts their health. Sample items: “My work has a positive influence on my health” and “My work has a negative influence on my health.” The items were made for the ARK study and have been published elsewhere (Innstrand et al., 2022; Langseth-Eide, 2019).

### Statistical analyses

All statistical analyses were performed using the structural equation modeling (SEM) package on Stata statistical software version 14.1 (Acock, 2013), with maximum likelihood (ML) and listwise deletion. Such restriction to complete cases only reduced the final analytic sample size to 10,096. We perform a Doornik-Hansen chi-square test to assess the joint normality of the observed variables (Doornik & Hansen, 2008), in which we reject the null hypothesis of joint normality (*p* < 0.001). To mitigate Type I error when using the ML estimator with non-normal data, we estimate all models using Satorra-Bentler robust standard errors and report both the ML and Satorra-Bentler chi-squared test statistics (Curran, West, & Finch, 1996; Maydeu-Olivares, 2017).

All the latent variables were allowed to correlate, as they all represent psychosocial work variables assumed to be related. As the chi-square statistic is found to be sensitive to large sample size (Mehmetoglu & Jakobsen, 2016) the SEM model was considered by these additional fit indices: Comparative Fit Index (CFI), Tucker-Lewis Index (TLI), Root Mean Square Error of Approximation (RMSEA) and the Standardized Root Mean Square Residual (SRMR). A value of 0.90 or above is considered an acceptable fit for the CFI and the TLI, and a value of 0.08 or lower for the RMSEA and the SRMR (Acock, 2013). The statistical analyses were performed in three steps.

First, we performed confirmatory factor analysis (CFA) to ensure that the measurement model appropriately fit the data. Fit indices were first computed for the measurement model under the assumption that all latent constructs (but no item-specific error terms) were correlated. To strengthen the model fit, we modified the measurement model consistent with the estimated modification indices to allow for correlation in the error terms between two pairs of items in the *work engagement* scale: First, item 1 (“At my work, I feel bursting with energy”) and item 2 (At my job, I feel strong and vigorous”), and second, item 4 (“I am immersed in my work”) and item 5 (“I get carried away when I’m working”).

To confirm each item predicted adequate variation in the intended underlying construct, we examined standardized factor loadings and average variances extracted (AVE) for each latent variable using a cutoff point of 0.5 for both (Mehmetoglu & Jakobsen, 2016). The unique contribution of each underlying construct (discriminant validity) was assessed by the magnitude of each factor’s AVE relative to the squared correlation (SC) of the other factors. By convention, if the AVE is greater than the SC for all other factors, we conclude that the factor is distinct from other constructs (Mehmetoglu & Jakobsen, 2016).

All standardized factor loadings were sufficiently large (>0.5) except for one task completion clarity item (“*I know when a task is completed*”), which was estimated to be 0.30. In an assessment of equation-level goodness of fit, this item had a corresponding *R*-squared value of <0.20; these two estimates together led us to remove this item.

**Figure 1.**
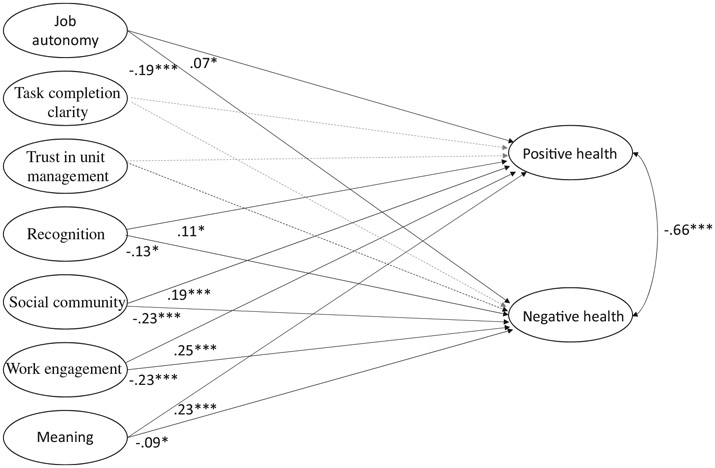
Structural model with standardized parameter estimates. *Note:* ***p < 0.001; *p < 0.05. Dotted line indicates non-significant relationship. *N* = 10,096.

Next, to assess which of the suggested psychosocial need variables are the strongest predictors for health, we estimated a full SEM model in which all seven latent constructs were regressed on positive and negative work-related health for the whole sample (see Figure 1). Including both outcomes in the same model allowed for simultaneous estimation of the pathways between the latent variables and positive and negative work-related health. As two distinct but closely related parts of work-related health, the error terms of the two outcome measures were allowed to correlate.

Finally, we performed multi-group CFA (MG-CFA) and multi-group SEM (MG-SEM) to examine potential subgroup differences. Specifically, we assessed whether the mean value of each of the seven needs, as well as its predictive value on the two health outcomes, differed significantly between gender and across the three employee groups: faculty, doctoral students (PhD), and technical/administrative staff. For each of the two sets of analyses, we estimated three subgroup models in which all parameters were allowed to vary (model 1), factor loadings were invariant (model 2), and both factor loadings and intercepts were invariant (model 3). In each model with imposed constraints, we performed Wald tests to assess whether the assumption of invariance is valid (StataCorp, 2017, p. 71) and likelihood-ratio tests to compare nested models.

## Results

### Measurement model

The goodness-of-fit estimates for the final measurement model all suggest a strong model fit: CFI = 0.96, TLI = 0.95, RMSEA = 0.05, and SRMR = 0.03. The standardized factor loadings were sufficiently large (>0.5) ranging from 0.60 to 0.93. As shown in Table 2, estimated AVEs for the seven factors ranged from 0.49 to 0.80. However, the comparison of the magnitude of each factor’s AVE relative to the squared correlation (SC) suggests a lack of discriminant validity between *meaning* and *work engagement* as well as between *trust in unit management* and *recognition*. As these factors were representing different aspects of the same need, the need for professional growth or positive emotions, and psychological safety, respectively, such a lack of discrepancy was somewhat expected. The estimated composite reliability (CR) measures ranged from 0.78 to 0.95, suggesting adequate reliability of the final measurement model (Mehmetoglu & Jakobsen, 2016).

### Full structural equation model

A full structural equation (SEM) model with all seven latent constructs were regressed on each of our outcome measures, positive and negative work-related health, provided a good fit: RMSEA = 0.05, SRMR = 0.03, CFI = 0.96, TLI = 0.95. The Satorra-Benlter-corrected fit statistics are nearly identical (RMSEA = 0.04, SRMR = 0.04, CFI = 0.96, TLI = 0.95), and the *p*-values for the ML and Satorra-Bentler likelihood ratio tests are both <0.001. As shown in Figure 1, all factor loadings, except task completion and trust in unit management (*ns*), were significantly related, and in the intended direction to positive and negative health.^2^ The three strongest relationships to positive health were work engagement (*β* = .25, *p* < 0.001), meaning (*β* = .23, *p* < 0.001), and social community (*β* = .19, *p* < 0.001). The three strongest relationships to negative work-related health were work engagement (*β* = −.23, *p* < 0.001), social community (*β* = −.23, *p* < 0.001), and autonomy (*β* = −.19, *p* < 0.001). In addition, recognition was positively related to positive work-related health (*β* = .11, *p* < 0.05) and negatively related to negative work-related health (*β* = −.13, *p* < 0.05). It should be noted that some of the antecedent variables were differentially related to positive and negative work-related health. For example, autonomy was weaker related to positive work-related health (*β* = .07, *p* < 0.05) as compared to negative work-related health (*β* = −.19, *p* < 0.001). Conversely, the meaning was more strongly related to positive work-related health (*β* = .23, *p* < 0.001) as compared to negative work-related health (*β* = −.09, *p* < 0.05). Thus, although these two health constructs were significantly related (*β* = −.66, *p* < 0.001), they are differentially related to psychosocial work variables.

### Multi-group analyses

#### Differences in mean levels of latent variables

To examine potential subgroup differences in the *level* of the needs, we performed multi-group CFA (MG-CFA) across gender and across the three employee groups (faculty, doctoral students (PhD), and technical/administrative staff).

In general, the single group solutions suggested a good model fit for both men and women, and across employee groups. The RMSEA ranged from .048 to .052, and the CFI and TLI were above the .90 threshold. Invariance of the measurement model was further tested by running MG-CFAs with three levels of constraints: (1) an unconstrained model, (2) equal factor loadings, and (3) equal factor loadings and intercepts. Although the chi-square increase was significant for each nested model in the test of factorial invariance across gender and faculty groups, the preceding models were no worse as the change in CFI did not exceed −.02 (Vandenberg & Lance, 2000), and the change of RMSEA was <.015 (Chen, 2007). Consequently, between-group differences in latent means could be calculated (Chen, 2008).

By constraining the factor loadings and intercepts to be equal across groups, we examined the mean differences in the level of needs (see Table 3 for means and standard deviations across subgroups groups). As required by latent mean analyses, the latent mean is fixed to zero in one group, called the reference group, and estimated in the comparison group (s). In the present study, females, and faculty members (research and teaching) were used as the reference group for the multi-group analyses on gender and employee groups, respectively. As can be seen in Table 4, male experience significantly more autonomy, recognition, task completion clarity, and trust in unit management, as compared to females, but also less social community. There were no gender differences in the mean level of meaning or work engagement. To better contextualize these statistically significant mean differences, we also calculate Cohen’s *d* statistics for each pairwise comparison. The Cohen’s *d* statistics for mean comparisons across genders range from 0.05 (task completion clarity) to 0.125 (autonomy), which suggests the differences are relatively small according to conventional thresholds (Sullivan & Feinn, 2012).

Regarding occupational differences, the doctoral students and the technical/administrative staff were significantly different from the faculty in all the mean levels of the proposed needs (Table 4). More specifically, the doctoral students and the technical/administrative staff reported more recognition, social community, and trust in unit management as compared to the faculty, but also less meaning, work engagement, and task completion clarity. The doctoral students reported a higher level of autonomy than the faculty, and the technical/administrative reported less. In terms of effect sizes, the largest mean difference was found in the reported meaning between faculty and technical administrative staff (Cohen’s *d* = 0.3). Meanwhile, the largest difference between faculty and doctoral students was reported in autonomy, although the Cohen’s *d* statistic (0.28) still suggests relatively small differences.

#### Structural model: testing for multi-group invariance

To examine potential subgroup differences in the *predictive value* of the needs of the two work-related health outcomes, we performed multi-group SEM (MG-SEM) across gender and across the three employee groups (faculty, doctoral students (PhD), and technical/administrative staff).

##### Gender.

A group sensitive model (unconstrained) for gender had a significantly better fit than a universal model (constrained) according to differences in the Chi-square value [Δχ^2^(*df*) = 103.5(36), *p* < 0.001]. The other fit indices were left unchanged. Thus, gender differences in the strength of the relationship in the hypothesized structural model can be assumed. More specifically, as can be seen in Table 5, the relationship between the needs and positive work-related health was significantly different across gender for autonomy, meaning, work engagement, and task completion clarity. In predicting negative work-related health, there were significant gender differences in autonomy and task completion. In one case (the effect of task completion clarity on negative work-related health), we found significant across-group differences as well as gender-specific effects that were significant in opposite directions (*B* = 0.06, 95% CI: 0.01, 0.11 among women, *B* = −0.08; 95% CI: −0.14, −0.03 among men). Among females, the strongest predictors of positive and negative work-related health were work engagement (*B* = 0.30; 95% CI: 0.24, 0.36) and autonomy (*B* = −0.27; 95% CI: −0.36, −0.18), respectively. Among males, the strongest predictors were meaning (*B* = 0.32; 95% CI: 0.21, 0.43) and social community (*B* = −0.24; 95% CI: −0.32, −0.17) for positive and negative work-related health, respectively.

##### Employee groups.

Similarly, a group sensitive model (unconstrained) for occupational groups, had a significantly better fit than a universal model (constrained) according to differences in the Chi-square value [Δχ^2^(*df*) = 367.5(72), *p* < 0.001]. The other fit indices were left unchanged. Thus, differences in the strength of the relationship in the hypothesized structural model could be assumed for the different occupational groups as well. However, as can be seen in Table 5, we found no evidence of significant occupational group differences in the relationship between the needs and positive health, and only one need (trust in management) differed across these groups with respect to negative work-related health. Specifically, the relationship between trust in management and negative work-related health among respondents in administrative/technical positions appeared to be stronger than the other two groups (*B* = −0.21; 95% CI: −0.34, −0.09). Among faculty and doctoral students, this association was not significant at *p* = 0.05.

## Discussion

Informed by the seven needs of importance for health (Maslach & Banks, 2017) and the “Essential Elements” for the twenty-first century faculty work (Gappa & Austin, 2010), the present study examined the predictive value of psychosocial needs for positive and negative work-related health in university settings. The study also examined group differences (gender and academic groups) in the proposed relationship, and in the level of these needs. We found evidence supporting positive relationships between autonomy, recognition, social community, meaning, work engagement, and positive work-related health, and supporting negative relationships with negative work-related health. This evidence supports most of the hypothesized relationships (H1a, H2a, H3b–c, H4b–c, H5a–b, and H6a–b). However, two needs, task completion clarity and trust in unit management were not related to either positive or negative work-related health (rejecting H1b, H2b, H3a, and H4a). Although these two needs were suggested to be of importance previously in an academic setting, they did not appear to affect work-related health positively or negatively in this sample. It should be noted that the lack of discriminant validity and high correlation (*r* = 0.87) between trust in unit management and recognition could indicate collinearity with recognition accounting for all the variance explained by trust in unit management. Nevertheless, this would not explain the lack of significant association between task completion clarity and positive/negative work-related health, which is not strongly correlated with other latent variables. Overall, our findings suggest that work engagement and social community are the strongest predictors of both positive *and* negative work-related health. Thus, any actions aiming to boost engagement in work and facilitate collegiality and community would be important steps toward a healthy university. This is in line with Gappa and Austin (2010), who argue that collegiality and professional growth are particularly important in times of change in academic work.

In contrast to previous findings regarding SDT suggesting that autonomy is more strongly related to general well-being than relatedness (Van den Broeck et al., 2016), our findings suggest that social community is more strongly related to both positive and negative work-related health than autonomy in this sample. Our findings also deviate from previous studies suggesting that SDT needs are more related to positive outcomes than negative outcomes (Van den Broeck et al., 2016). On the contrary, our findings show that lower levels of autonomy and social community (relatedness) are more strongly related to negative work-related health. Even though the two work-related health measures were highly correlated, some variables showed differential relationships with positive and negative work-related health, reinforcing the appropriateness of measuring positive and negative work-related health independently. Whereas autonomy was more strongly related to negative work-related health, meaning was a better predictor for positive work-related health. This suggests that the mechanisms underlying health promotion are likely to differ from those that detract from health prevent illness. Thus, this implies that if autonomy is undermined significantly in the university setting, harmful effects on faculty health may result. In addition, promotion of the meaningfulness of work is essential for enhancing health and well-being. This result is bolstered by a qualitative study which explored the experience of meaningful work in three occupations (refuse collectors, stonemasons, and faculty) and found that the strongest experience of meaningfulness arose during shared rituals or ceremonies held to mark the completion of a piece of work (Bailey & Madden, 2017). For the faculty surveyed in this study, these experiences may have included presenting their work at a conference, giving a well-received lecture, or seeing a doctoral student graduate successfully.

Hypothesis 7, which tested whether the level of the psychosocial needs would be variant (H7a) and that the strength of the association between the seven needs and positive/negative work-related health would be invariant (H7b), was partly supported for gender. Males reported higher levels of autonomy, recognition, task completion clarity, and trust in unit management and less social community compared to females, although we note that the magnitude of these differences is relatively small. Nonetheless, the latter finding is consistent with a meta-analytic analysis suggesting that females experience more relatedness than males (Van den Broeck et al., 2016). There were no gender differences in the mean level of meaning or work engagement.

There were significant differences between males and females in which factors influenced their work-related health positively. Males appeared to emphasize the influence of meaning, whereas females emphasized the influence of work engagement more strongly. As females report significantly lower levels of autonomy compared to males, and low levels of autonomy predict strongly negative work-related health for females, organizations should actively reinforce autonomy to boost females’ health and well-being. For males, significantly lower levels of social community exerted a strong negative influence on their work-related health. Our study results suggest that efforts to increase feelings of being a part of a social community for males would be beneficial for their health in the long run.

Both males and females reported a relationship between task completion clarity and negative work-related health. However, males reported that greater task completion clarity lowered their perception that the work negatively impacted their health, whereas females reported the opposite. That is, greater task completion clarity increased the female’s perception that the work negatively impacted their health. Moreover, for the women greater task completion clarity was associated with a decrease in the assumption that work positively impacts their health. As suggested by Van den Broeck et al. (2016), the determination of when a task is completed does not guarantee that one has mastered the task. Another explanation for this unexpected result relates to the duality of autonomy and the mixed findings linking autonomy with mental health (Maslach & Banks, 2017). Although Norway has the highest proportion of females employed (45%) in higher education within Europe, the European Commission’s 2012 meta-analysis of gender and science research (Caprile et al., 2012) found that female advancement in science was slow compared to males and being a minority member in a male-dominated occupation might cause females to feel they need to prove their competence in a broader sense and be more conservative in deciding when a task is adequately performed. This difference in the perception of task completion could nurture a boundaryless work life for females with serious consequences for their health. Indeed, work-family issues have been found to be the highest ranked problem for females in academia (McGuire, Bergen, & Polan, 2004), and the strongest reason for females who consider leaving academic medicine (Foster et al., 2000). As the female advancement in science is slow compared to males, academic levels (e.g., Professor, vs. Associate Professor, vs. Assistant Professor) could also be a potential confounding variable. Thus, the gender differences found in the present study may not be ascribed to gender per se but to gender differences in academic levels. Thus, we recommend future studies to explore gender differences in association with their academic level.

The multi-group analyses across the three academic groups (faculty, doctoral students, and technical/administrative staff) mainly supported hypothesis seven, as we found significant differences in psychosocial need levels across these groups (H7a) but their relationship to positive/negative work-related health was similar (H7b). An exception was trust in unit management, which showed a negative influence on negative work-related health among technical/administrative staff but was not significant for faculty and doctoral students. Doctoral students and technical/administrative staff reported more recognition, social community, and trust in unit management but less meaning, work engagement, and task completion clarity compared to the faculty. Doctoral students reported a higher level of autonomy than the faculty whereas the technical/administrative staff reported less. These level differences could be attributed to differences in the tasks performed across employee groups, resulting in different degrees of opportunity for need satisfaction. Again, we note that the magnitude of the mean differences is relatively small (albeit statistically significant), but given these need’s strong association with work-related health, these differences across groups are qualitatively meaningful.

### Limitations

The study’s strength is its large sample and the use of sophisticated statistical analyses, which enable more sensitive analyses of group differences compared to traditional statistical techniques like ANOVAs and *t*-tests (Hong, Malik, & Lee, 2003). The findings also need to be interpreted with limitations. First, the study was based on the needs theory (Maslach & Banks, 2017) and previous findings of “Essential Elements” for the twenty-first century faculty work (Gappa & Austin, 2010). Although our seven psychosocial variables related to these findings, they do not directly represent need satisfaction in the same way as variables studied in SDT (Deci & Ryan, 2000). Rather, our variables were proxies for the psychosocial needs presumed to be important for university employees. Additionally, we were not able to measure competence, one of the three basic needs assumed by SDT, within our dataset.

Our measure of health may also restrict a thorough examination of the relationship between need satisfaction and health. Although self-rated, single item measure of health has proved to be a reliable measure for health (DeSalvo, Bloser, Reynolds, He, & Muntner, 2006), and is judged to be appropriate for use in population surveys in general, and in particular, when used as an outcome variable to avoid overlap with different multi-item predictors (Bowling, 2005), multi-item measures are less prone to socio-psychological biases (Bowling, 2005), and the study results should be interpreted with this in mind. Future studies could benefit from using clinical measures of health. It should be noted that although job and life satisfaction is closely related, the health measure used in this study was restricted to factors at work. The rationale for this was to sort out factors like a broken leg due to skiing or other non-work-related factors. However, we recognize the interdependent relationship between well-being at work and well-being at life in terms of work meeting psychosocial and other needs and as a potential explanation for gender differences.

A second issue relates to cross-sectional designs as they preclude the possibility of drawing causal conclusions. Although SEM analysis and large samples compensate for much of the concerns related to these designs, the present findings can only be seen as significant relationships. Finally, the present sample consists of university employees only. Hence, the present findings can only be generalized to this occupational group.

### Conclusion

The findings in the present study confirm the universality of the effect of needs on health across different employee groups despite differences in the level of the needs and are consistent with SDT (Deci & Ryan, 2000), and previous findings (Chen et al., 2015). Moreover, Essential Elements seem to generalize across university employees as suggested by Gappa and Austin (2010). Males and females, on the other hand, appear to differ both in how they perceive the influence of these needs on their health, as well as in the character of these needs. Future studies should confirm these findings by testing these assumptions on different samples and with a longitudinal design, more clinical measures on health, and SDT approved variables. Nevertheless, as suggested by the present study, universities may achieve greater equality by considering individual variances in the sources of need satisfaction in constructing health-promoting initiatives to create a healthy university for all.

## Figures and Tables

**Table 1.** Demographic characteristics and employment conditions for the three different employee groups.

	Faculty (*n* = 4562)		PhD students (*n* = 1452)		Admin/technical (*n* = 5519)	
	*n*	%	*n*	%	*n*	%
Gender						
Women	2047	44.9	772	53.2	3443	62.4
Men	2514	55.1	680	46.8	2076	37.6
Age category						
←29 years	103	2.3	656	45.6	415	7.6
30–39 years	891	19.8	567	39.4	1309	23.9
40–49 years	1249	27.8	176	12.2	1645	30.1
50–59 years	1267	28.2	36	2.5	1348	24.6
60 years →	982	21.9	3	.2	752	13.8
Terms of employment						
Permanent employee	3463	77.0	140	9.7	4846	89.8
Temporary employee	1032	23.0	1299	90.3	552	10.2
Position						
1–50%	217	4.8	4	.3	60	1.1
51–99%	523	11.5	114	7.9	745	13.5
Full time (100%)	3820	83.8	1334	91.9	4714	85.4

*n*: frequency; %: valid percent.

**Table 2.** Means, standard deviation (*SD*), average variance extracted (AVE), and squared correlations of latent variables (SC).

Variable	Mean	*SD*	1	2	3	4	5	6	7
1. Autonomy	3.84	.64	–						
2. Meaning	4.01	.68	0.38	–					
3. Work engagement	4.58	1.05	0.21	0.63	–				
4. Recognition	3.79	.85	0.44	0.27	0.16	–			
5. Social community	3.97	.77	0.31	0.25	0.16	0.42	–		
6. Task completion clarity	3.58	.79	0.28	0.06	0.04	0.04	0.26	–	
7. Trust in management	3.71	.91	0.35	0.19	0.13	0.87	0.36	0.29	–
AVE	–	–	0.49	0.62	0.59	0.73	0.64	0.64	0.80
CR	–	–	0.78	0.83	0.93	0.89	0.84	0.78	0.95

AVE: average variance extracted; CR: composite reliability.

*Note: N* = 10,096.

**Table 3.** Means and standard deviation (*SD*) for latent variables for all groups, by gender and employee group.

	Female		Male		Academic		PhD students		Technical/administrative	
	Mean	*SD*	Mean	*SD*	Mean	*SD*	Mean	*SD*	Mean	*SD*
Autonomy	3.81	.64	3.89	.64	3.86	.65	4.03	.58	3.77	.63
Meaning	4.01	.67	4.01	.68	4.12	.63	4.00	.75	3.92	.68
Work engagement	4.60	1.05	4.56	1.05	4.72	.94	4.55	1.02	4.47	1.12
Recognition	3.76	.85	3.82	.85	3.74	.90	3.81	.74	3.83	.84
Social community	4.00	.76	3.94	.77	3.87	.80	3.93	.75	4.06	.73
Task completion clarity	3.57	.81	3.61	.77	3.67	.76	3.49	.81	3.54	.81
Trust in management	3.68	.92	3.76	.91	3.64	.97	3.82	.79	3.74	.90

*Note: N* = 10,096.

**Table 4.** Gender and employee group differences in the mean level of the psychological needs (MG-CFA).

Group	Autonomy	Meaning	Work engagement	Recognition	Social community	Task completion clarity	Trust in unit management
Gender							
Female^R^	0	0	0	0	0	0	0
Male	0.09***	0.01	−0.01	0.06***	−0.04**	0.04*	0.07***
Employee group							
Faculty^R^	0	0	0	0	0	0	0
PhD	0.20***	−0.11***	−0.13***	0.08**	0.08***	−0.17***	0.18***
Techn/admin	−0.10***	−0.20***	−0.19***	0.08***	0.18***	−0.12***	0.10***

*Note:* Stars refer to significant group differences as indicated by Wald-test for group invariance. ^R^Reference group. ***p < 0.001; **p < 0.01; *p < 0.05. *N* = 10,153 and *N* = 10,154 for gender and employee group models, respectively.

**Table 5.** Path coefficients of the multi-group structural equation model (MG-SEM), by gender and employee group.

	Gender		Employee group		
	Female *B* (95% CI)	Male *B* (95% CI)	Faculty *B* (95% CI)	PhD student *B* (95% CI)	Admin/Technical *B* (95% CI)
Positive work related health					
Autonomy	0.19*** **(0.10, 0.27)**	**−0.06 (−0.15, 0.03)**	0.15** (0.06, 0.25)	−0.03 (−0.21, 0.15)	0.13* (0.03, 0.23)
Meaning	0.16*** **(0.07, 0.26)**	0.32*** **(0.21, 0.43)**	0.26*** (0.14, 0.38)	0.24* (0.05, 0.43)	0.23*** (0.12, 0.34)
Work engagement	0.30*** **(0.24, 0.36)**	0.19*** **(0.13, 0.26)**	0.21*** (0.15, 0.27)	0.31*** (0.17, 0.45)	0.26*** (0.21, 0.32)
Recognition	0.10 (−0.03, 0.23)	0.11 (−0.04, 0.26)	0.10 (−0.05, 0.24)	0.32 (−0.15, 0.80)	0.00 (−0.14, 0.14)
Social community	0.18*** (0.13, 0.23)	0.20*** (0.14, 0.26)	0.19*** (0.13, 0.26)	0.03 (−0.10, 0.17)	0.16*** (0.11, 0.22)
Task completion	−0.06*** **(−0.10, −0.02)**	**0.05 (−0.00, 0.10)**	−0.04 (−0.10, 0.02)	0.02 (−0.08, 0.12)	−0.03 (−0.08, 0.02)
Trust in management	0.06 (−0.05, 0.16)	0.10 (−0.02, 0.22)	0.03 (0.05, 0.27)	−0.04 (−0.43, 0.34)	0.16** (0.51, 0.27)
Negative work related health					
Autonomy	−0.27*** **(−0.36, −0.18)**	**−0.08 (−0.18, −0.02)**	−0.30*** (−0.40, −0.19)	−0.12 (−0.32, 0.09)	−0.24*** (−0.36, −0.23)
Meaning	−0.07 (−0.17, 0.04)	0.11 (−0.24, 0.01)	−0.14* (−0.32, 0.09)	−0.06 (−0.28, 0.15)	−0.06 (−0.18, 0.07)
Work engagement	−0.26*** (−0.32, −0.20)	−0.21*** (−0.28, −0.14)	−0.19*** (−0.26, −0.12)	−0.38*** (−0.54, −0.23)	−0.24*** (−0.30, −0.17)
Recognition	−0.04 (−0.19, 0.10)	−0.22* (−0.40, −0.05)	−0.09 (−0.26, 0.07)	−0.65* (−1.20, −0.11)	0.01 (−0.15, 0.17)
Social community	−0.23*** (−0.29, −0.17)	−0.24*** (−0.32, −0.17)	−0.21*** (−0.29, −0.13)	−0.02 (−0.18, 0.13)	−0.22*** (−0.28, −0.16)
Task completion	**0.06 (0.01, 0.11)**	−0.08** **(−0.14, −0.03)**	0.21 (−0.04, 0.09)	−0.23 (−0.14, 0.09)	0.01 (−0.04, 0.07)
Trust in management	−0.14* (−0.25, −0.02)	−0.02 (−0.16,0.11)	**−0.05 (−0.18, 0.08)**	**0.29 (−0.15, 0.74)**	−0.21** **(−0.34, −0.09)**
Observations	5446	4650	3972	1267	4858

*Note:* ***p < 0.001; **p < 0.01; *p < 0.05.

Bold text indicates significant group differences as indicated by Wald-test for group invariance (*p* < 0.05).

## Footnotes

1. Norwegian acronym for work environment and climate study.

2. To account for potential bias introduced by listwise deletion, we also estimate our main SEM using the MLMV estimator in Stata, which accounts for missingness. Our results are unchanged when using this estimator with two notable exceptions. First, trust in unit management is a statistically significant predictor of positive (*β* = 0.08, *p* < 0.05) and negative (*β* = −0.11, *p* < 0.05) work-related health, while recognition is no longer a statistically significant predictor of negative health. Full results available upon request.
